# Exploring pre-pandemic patterns of vaccine decision-making with the 5C model: results from representative surveys in 2016 and 2018

**DOI:** 10.1186/s12889-024-18674-9

**Published:** 2024-04-30

**Authors:** Sarah Eitze, Lisa Felgendreff, Nina Horstkötter, Linda Seefeld, Cornelia Betsch

**Affiliations:** 1 Institute for Planetary Health Behaviour, University Erfurt, 99089 Erfurt, Germany; 2 Health Communication Working Group, Implementation Research, Bernhard Nocht Institute for Tropical Medicine, University of Hamburg, Hamburg, Germany; 3 Department of Journalism and Communication Research, Hannover University of Music, Drama, and Media, Hanover, Germany; 4 Federal Centre for Health Education, Cologne, Germany

**Keywords:** Vaccination intentions, Vaccination behaviour, 5C model, Health decision-making, Vaccine hesitancy

## Abstract

**Background:**

The 5C psychological antecedents of vaccination (Confidence, Complacency, Constraints, Calculation, and Collective Responsibility) facilitate understanding vaccination decisions in specific target groups as well as the general public’s informational needs. This study aims to explain pre-pandemic vaccination behaviour (a) in general, (b) for specific vaccines such as influenza, and (c) for certain target groups (e.g. people over the age of 59 years, parents, healthcare workers), using the 5C model and sociodemographic variables. The intention to get an influenza vaccination was also analysed for target groups.

**Methods:**

The 5C, self-reported vaccination behaviour and the intention to vaccinate were collected in two representative telephone surveys in Germany – one in 2016 (n_1_ = 5,012) and another in 2018 (n_2_ = 5,054). Parents, people over the age of 59 years, chronically ill people, people with a migratory background, pregnant women and healthcare workers were target groups.

**Results:**

Overall, the 5C model had higher explanatory power than sociodemographic variables. The pattern of vaccine hesitancy slightly differed between vaccinations and target groups. Confidence in safety and effectiveness was always a significant predictor. Complacency (the underestimation of disease risks) and Constraints were significant predictors as well. Calculation (of risks and benefits) was important for influenza vaccination intentions.

**Conclusions:**

This work builds an important benchmark for understanding potential changes in vaccine acceptance due to the pandemic. The benchmark can be used in research on potential effects of the pandemic on vaccination behaviours. Intervention designers can also use the results to understand specific audiences and their vaccination decisions.

**Supplementary Information:**

The online version contains supplementary material available at 10.1186/s12889-024-18674-9.

Monitoring vaccination behaviour and understanding its antecedents are crucial for containing infectious diseases [1, 2]. The problem of vaccine hesitancy is so widespread that, in 2019, the WHO listed it as one of the top 10 threats to global health [3]. Even in times of a global pandemic and a resurgence of measles, there is still serious vaccine hesitancy resulting in insufficient vaccine uptake in many countries. One reason may be that people integrate negative attitudes from prior vaccination decisions such that missed opportunities of vaccination (MOVs) increase [4]. A MOV refers to any contact with health services by an individual (child or person of any age) who is eligible for vaccination but refuses to get vaccinated [5]. It is therefore crucial to understand what affected vaccination behaviour before these crises emerged. In addition, having a clear benchmark and knowledge about what affected vaccination behaviours before the pandemic will help to better understand the potential changes and pathways of change. The main purpose of this article is therefore to draw a map of pre-pandemic vaccination decisions in different target groups to understand their sociodemographic and psychological determinants of vaccination. The data were collected as part of the Infection Protection Study in the years 2016 and 2018 [6, 7]. The study is designed as cross-sectional with biennial monitoring, and the results are made available to the public and policymakers through descriptive reports.

Vaccination is an individual health decision [8]. Depending on their decisions, individuals can be placed on a continuum from denial to acceptance of vaccination in general [8] and of specific vaccines, such as the influenza vaccine [9]. Besides demographic antecedents like age, gender, education, and individual characteristics such as chronic illnesses [9, 10], psychological antecedents can be explored with the 5C model [11]. With this validated and globally used scale, five psychological antecedents of vaccination intention and behaviour can be investigated for research and health intervention design (Fig. 1). *Confidence* includes high trust in the effectiveness and safety of vaccines as well as in the system delivering and recommending the vaccines. *Complacency* is defined as a lack of risk perception for diseases that vaccines can prevent. *Constraints* are perceived barriers to vaccination, such as possible costs and time investments. *Calculation* is conceptualised as the need for a personal risk–benefit analysis, in which individuals often extensively search for information. *Collective Responsibility* describes the importance and awareness of herd immunity as an achievable goal of vaccination. In contrast, low Collective Responsibility would predict free-riding on others’ vaccination behaviour. Taken together, the 5Cs are highly relevant to the vaccination decision and explain up to 80% of the variance in regressions that explain differences in general as well as vaccine-specific vaccination intentions [11].

**Fig. 1:**
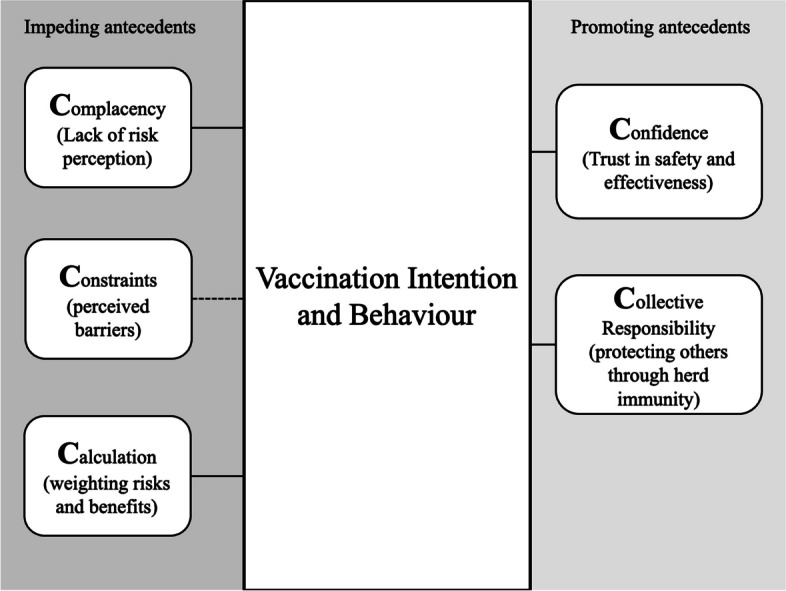
Overview of the 5C antecedents. *Note.* Higher Confidence [9, 12] and Collective Responsibility [6, 13] are related to higher vaccination intention and behaviour. Higher Complacency [14] and Calculation [15, 16] are related to lower vaccination intention and behaviour. Constraints are related to lower vaccination behaviour [17], but with intentions, the results are inconclusive [11]

This study explored the influence of demographic and psychological antecedents on vaccination intention and behaviour (a) over cross-sectional samples from different years (b) across different vaccines and (c) across six target groups. The results show a pattern of vaccination hesitancy before the COVID-19 pandemic in Germany. The data will serve as the foundation needed to understand possible secondary pandemic effects on vaccination behaviour and intention, such as on influenza and general demand, in six target groups. The research question guiding the following analyses therefore was *“How are the sociodemographic and psychological (5C) antecedents associated with vaccination decisions within target groups in the German population?”.*

## Method

The German research institute ‘forsa.’ conducted the survey for the Centre for Federal Centre for Health Education in 2016 between 26 July and 18 September and in 2018 between 24 July and 12 September via computer-assisted telephone interviews (CATI method with random digit dialing) in a dual frame design (calling landline and mobile phone numbers) [6, 7]. Inclusion criteria were age (between 16 and 85 years) and the ability to participate in German. If a potential participant was unable to attend, they were called back at an agreed time. If no one picked up or was willing to participate, a new number was dialled. The dropout rates for landline and mobile phone calls differed in both years (telephone vs. mobile phone in 2016: 50.1% vs. 61.6%; 2018: 51.1% vs. 61.6%).

During the times of data collection, the vaccination schedule in Germany was target group–specific. In general, vaccination (against influenza, tetanus, etc.) in Germany is free of charge when officially recommended, except for very specific travel vaccinations such as rabies or dengue. Recommendations for the annual influenza vaccination are given for risk groups such as people over the age of 59 years, chronically ill people, or healthcare workers in contact with patients. Vaccination appointments are usually made with general practitioners; specialists in practices or hospitals also vaccinate.

### Participants

The total samples included *n*_2016_ = 5,012 and *n*_2018_ = 5,054 participants. Table 1 presents the demographic distribution. Target groups are not mutually exclusive (e.g. participants can be both parents and healthcare workers). The target groups were people over the age of 59 years, people with a chronic illness (people who reported having a chronic illness like chronic obstructive pulmonary disease (COPD), cardiovascular diseases or neurological diseases), people with a migratory background (following the EU definition as themselves or at least one parent not born in Germany), people working in healthcare (defined as every medical job including contact with patients), parents (having at least one child under the age of 18 years and living in their household), and pregnant women. Parents were asked about their own vaccination intentions and behaviour, followed by vaccination intentions and behaviours for their children, but these results will be reported elsewhere. Participation was not incentivised.

**Table 1:** Demographic and subgroup distributions

		**Total**	**2016**	**2018**
**Subgroup distribution**		*N* = 10,066	*N* = 5,012	*N* = 5,054
Age > 59 (years)	Under 60	7,136 (71.1%)	3,209 (64%)	3,927 (77.7%)
	60 +	2,907 (28.9%)	1,790 (35.7%)	1,117 (22.1%)
Chronic illness	Yes	3,223 (32.1%)	1,711 (34.1%)	1,512 (29.9%)
	No	6,811 (67.9%)	3,284 (65.5%)	3,527 (69.8%)
Working in healthcare	Yes	956 (14.5%)	456 (15.4%)	500 (13.7%)
	No	5,657 (85.5%)	2,503 (84.6%)	3,154 (86.3%)
	Missing data	3,453	2,053	1,400
Parent	Yes, at least one child	2,152 (21.4%)	1,092 (21.8%)	1,060 (21.0%)
	No	7,914 (78.6%)	3,920 (78.2%)	3,994 (79.0%)
Pregnancy	Yes, currently pregnant	1,003 (33.1%)	502 (35.3%)	501 (31.2%)
	No	2,027 (66.9%)	922 (64.7%)	1,105 (68.8%)
	Missing data	7,036	3,588	3,448
Born after 1970 and not immunised against measles	Yes	578 (5.7%)	253 (5%)	325 (6.4%)
	No	9,488 (94.3%)	4,759 (95%)	4,729 (93.6%)
Migratory background	Yes	719 (7.1%)	352 (7%)	367 (7.3%)
	No	9,347 (92.9%)	4,460 (93%)	4,687 (92.7%)
**Demographic distribution**		**Total**	**2016**	**2018**
Age (years)	Min: 16 Max: 84	*M* = 48.34 *SD* = 17.8	*M* = 50.74 *SD* = 18.1	*M* = 45.96 *SD* = 17.1
Gender	Female	5,949 (59.1%)	3,055 (61.0%)	2,894 (57.3%)
	Male	4,117 (40.9%)	1,957 (39.0%)	2,160 (42.7%)
Education	Low	3,757 (39%)	2,021 (42.3%)	1,736 (35.7%)
	Medium	2,493 (25.9%)	1,169 (24.4%)	1,324 (27.3%)
	High	3,388 (35.2%)	1,592 (33.3%)	1,796 (37.0%)
East/West residence	East	1,514 (15.8%)	816 (17.1%)	698 (14.5%)
	West	8,059 (84.2%)	3,957 (82.9%)	4,102 (85.5%)

Distribution of demographic variables over the two survey samples (2016, 2018) and for the complete analyses of the two consecutive surveys. Compared to 2016, the sample of 2018 was slightly younger, *t*(10,041) = 13.605, *p* < 0.001. The distribution of male and female participants was more balanced in 2018, *x* ^2^(2) = 14.039, *p* > 0.001. Respondents with higher education *x* ^2^(2) = 42.974, *p* < 0.001 and western German origin *x* ^2^(2) = 11.539, *p* < 0.001 were also significantly more common in 2018. Systematic omissions due to filtered questions are displayed where needed. Differences from the single analyses might have occurred due to missing data on the dependent variables. Even though the screening criteria for age were 16–85, the descriptive maximum age was 84 in the sample

### Measures

The English translations of all variables used in this analysis can be found in Supplementary Table S1. Participants provided extensive sociodemographic information about themselves (age, gender, education, urban/rural residential area, marital status, work in healthcare, children, migratory background, federal state, job status) as well as selected information on their partners, parents, and children as well as on chronic diseases. Education was recoded into low, middle, or high according to the International Standard Classification of Education [18]. For the variable of East/West German residence, federal states originating from the former German Democratic Republic were coded as 1 (East) as the reference category, and the pre-existing Federal States of the Federal German Republic were coded as 2. We excluded citizens from Berlin from all analyses due to its inner separation between East and West Germany (*n*_2016_ = 239, *n*_2018_ = 254). The 5C antecedents of vaccination were rated on a 5-point Likert scale (1 = strongly disagree to 5 = strongly agree; [11]). In 2016, participants answered the full 5C scale (15 items), while in 2018, they answered the 5C short scale (5 items). To allow for comparing the data from 2016 and 2018, we only used the overlapping items of the 5C short scale for the regression analyses. The item for Collective Responsibility (‘If everyone is vaccinated, I do not have to get vaccinated, too’) was recoded so that higher values indicate higher Collective Responsibility.

The dependent variables were self-reported vaccination behaviour and intention to receive selected vaccines themselves: All participants stated whether they had chosen not to be vaccinated (MOVs) and if they had received any vaccine (vaccination behaviour) in the last five years. If they had received any vaccine during this time, they specified whether it was against tetanus, pertussis, measles, varicella, rubella, or influenza. For each vaccine, there was a yes/no question. Furthermore, participants reported their influenza vaccination intention for the upcoming season. Individuals with a chronic disease, older than 59 years, or working in the medical sector were asked if they get vaccinated against influenza annually. All items were answered with ‘yes’, ‘no’, or ‘don’t know’. If participants did not know or refused to answer, they were excluded from the specific analyses.

### Statistical analyses

All analyses were conducted with R Studio (Version 3.6.3). Vaccination behaviour and intention were analysed for the whole sample and the subgroups. Analyses for annual influenza vaccination behaviour and vaccination intention for the upcoming influenza season were restricted to participants over 59 years of age, chronically ill individuals, and healthcare workers. We conducted a logistic regression with the sociodemographic variables and the 5C antecedents as predictors. The sociodemographic variables were age, gender, education (dummy coded categorical variable), East/West residence (East vs. West), and year of participation (2016 vs. 2018).

## Results

**Fig. 2:**
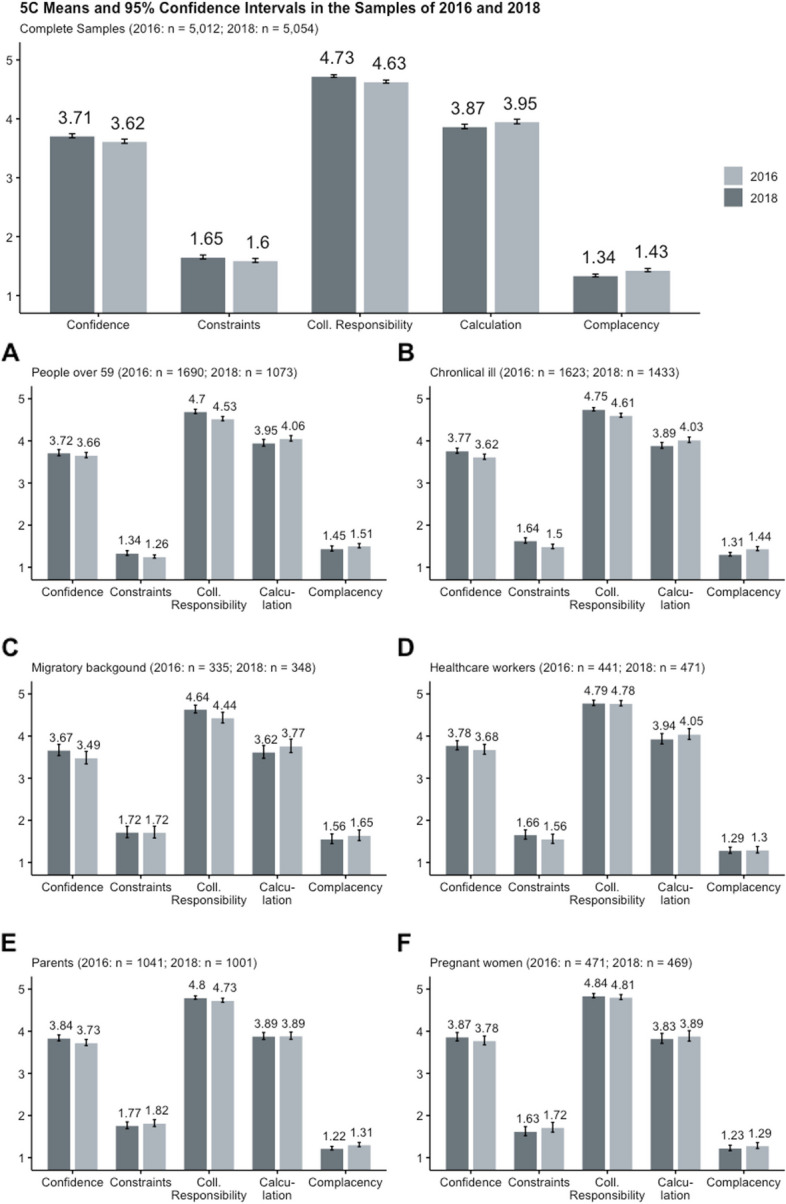
5C means and 95% confidence intervals in the samples. *Note.* The 5C values were marginally different between the different target groups in the samples of 2016 and 2018. Confidence was highest in parents (**E**), pregnant women (**F**), and healthcare workers (**D**). Constraints were highest in people with a migratory background (**C**) and parents (**E**). Collective Responsibility was higher in the 2018 sample compared to the 2016 sample. Calculation values were also above the scales’ average, showing the need for information regarding vaccination even before the COVID-19 pandemic and respective vaccination campaign. People with a migratory background had the lowest values for calculation. Complacency, i.e. neglecting disease risks, was highest in the sample of people with a migratory background (**C**) and in people over the age of 59 years (**A**)

The data and syntax of the following analyses are available in the OSF-Repository [https://osf.io/ezg2k/?view_only=58be5993ecda485baf803d1180803560]. Supplements S2–S6 show the tables for the regression analyses, and Supplements S7–S11 show the respective correlational tables. Figure 2 shows descriptive means and their 95% confidence intervals for the 5Cs in the various target groups.

### Missed opportunities for vaccination

**Fig. 3:**
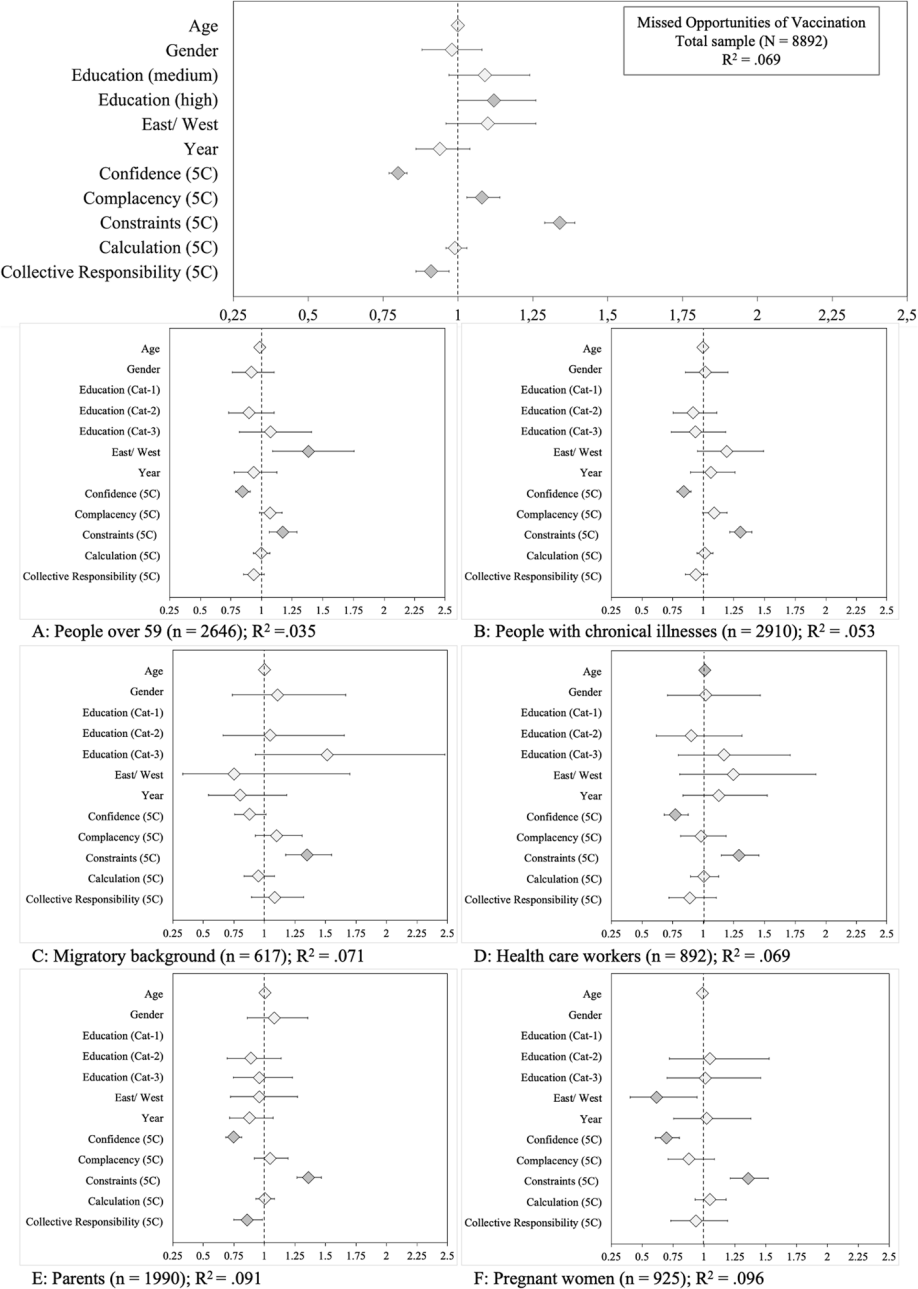
Results of regressions predicting missed opportunities for vaccination (‘In the last five years, have you declined an offer to get vaccinated?’). *Note.* Diamonds indicate the odds ratios and whiskers the 95% confidence intervals. Dark grey diamonds mark statistically significant predictors (*p* < 0.05). Gender (reference category: male), education (low), East/West (East), and year (2016) are dummy-coded variables. Gender was not entered in the logistic regression for pregnant women (**F**)

We evaluated whether the 5Cs are associated with previous vaccination decisions in the target groups. Overall, a quarter of the samples in 2016 (27.3%, *n* = 1,358) and 2018 (26.3%, *n* = 1,327) reported that they had at least once decided against a vaccination during the previous five years, which we use as an indicator for MOV. We investigated potential differences in antecedents of MOV for all subgroups. Supplementary Table S2 displays the logistic regression results, and Fig. 3 shows an overview of the odds ratios of the predictors per analysis. As can be seen in Fig. 3, antecedents associated with an increased probability of MOV in the total sample were low Confidence, high Complacency, high Constraints, and low Collective Responsibility. Of the sociodemographic variables, only higher (compared to lower) education was significantly related to MOV. Within the subgroups, patterns varied slightly. For people over 59 years of age, West-German residency was an additional predictor of MOV, whereas pregnant women living in East Germany were more likely to report not getting vaccinated. Particular attention should be paid to the finding that in the target group of people with a migratory background, only Constraints predicted MOV. Moreover, for healthcare workers, it was remarkable that next to Constraints, a lack of Confidence explained MOV.

### Vaccination behaviour

#### Previous vaccination behaviour

**Fig. 4:**
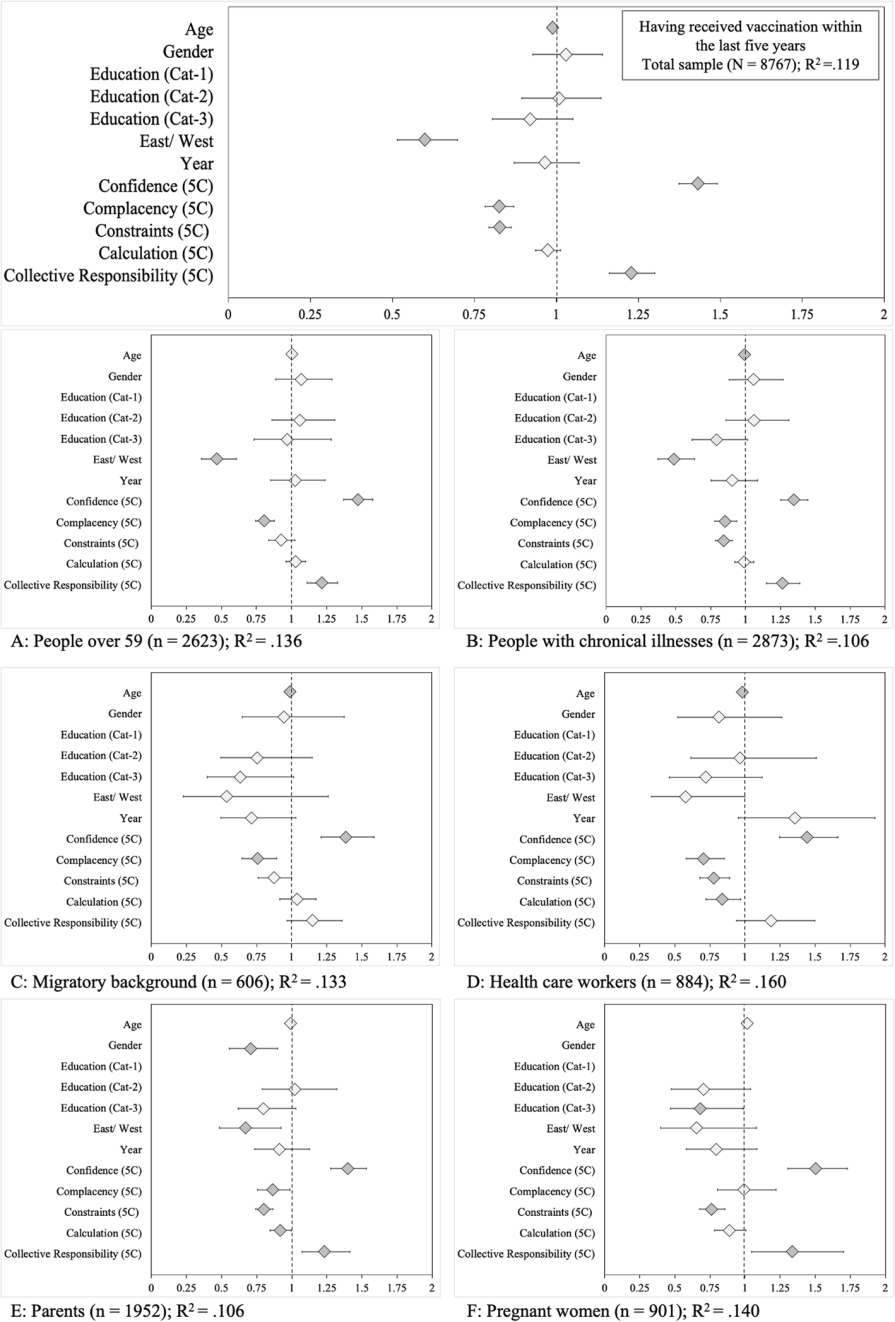
Antecedents of having received vaccination (‘Did you receive a vaccination in the last five years (since the summer of 2011/2013)?’). *Note.* Diamonds indicate the odds ratios and whiskers the 95% confidence intervals. Dark grey diamonds mark statistically significant predictors (*p* < 0.05). Gender (reference category: male), education (low), East/West (East), and year (2016) are dummy-coded variables. Gender was not entered in the logistic regression of the subgroup (F) pregnant women

Overall, about two thirds of the participants reported having received a vaccine during the last five years (prior to 2016: 68.7%, *n* = 3,440; prior to 2018: 68.6%, *n* = 3,466). Detailed results of the logistic regressions are displayed in Fig. 4, and values can be found in Supplementary Table S3. In the total sample, the predictors of having received a vaccination were lower age, East German residence, higher Confidence, lower Complacency and Constraints, and higher Collective Responsibility. In the subgroup analyses, there were differential effects. In the subgroup of parents, all 5C psychological antecedents of vaccination predicted having received vaccination. Fathers had received vaccinations more often than mothers had. For pregnant women, Constraints reduced the likelihood of having received vaccination in the past five years.

#### Seasonal influenza vaccination

In Germany, the seasonal influenza vaccination is officially recommended for risk groups, which include people over 59 years of age, people with a chronic illness, pregnant women, and healthcare workers [19].

##### Previous influenza vaccination behaviour

**Fig. 5:**
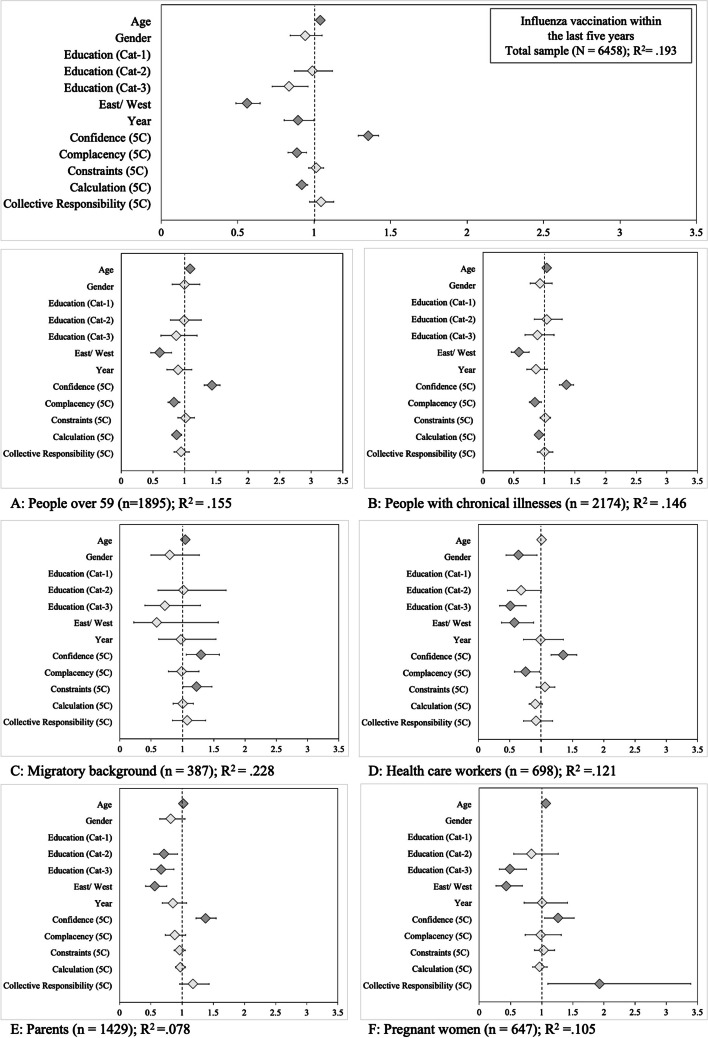
Previous influenza vaccination behaviour (‘Which of the following diseases have you been vaccinated against in the last five years? [Influenza: yes]’). *Note.* Diamonds indicate the odds ratios and whiskers 95% confidence intervals. Dark grey diamonds mark statistically significant predictors (*p* < 0.05). Gender (reference category: male), education (low), East/West (East), and year (2016) are dummy-coded variables. Gender was not entered in the logistic regression of the subgroup (**F**) pregnant women

Fewer participants reported having received an influenza vaccination within the last five years in 2018 (31.3%, *n* = 1,571) than in 2016 (35.2%, *n* = 1,777). Figure 5 and Supplementary Table S4 show the results of the logistic regressions for the total sample and the subsamples. The analyses of the full sample revealed that having received the annual influenza vaccination was more likely at a higher age and for East German residency. Medium (vs. low) education level was related to lower probability of influenza vaccination. For the 5C antecedents of vaccination, individuals with higher Confidence were more likely to have received the annual influenza vaccination, whereas highly complacent individuals and those calculating the pros and cons of vaccination were less likely. Within the subgroup analyses, three findings deviated from the general pattern. First, among healthcare workers, women were less likely than men to have received influenza vaccination. Among pregnant women, those with higher Collective Responsibility were more likely to have been vaccinated against influenza in the past five years. Finally, among individuals with a migratory background, greater Constraints were related to more influenza vaccination behaviour, which is contrary to the general theoretical assumptions about Constraints.

##### Annual influenza vaccination of risk groups

**Fig. 6:**
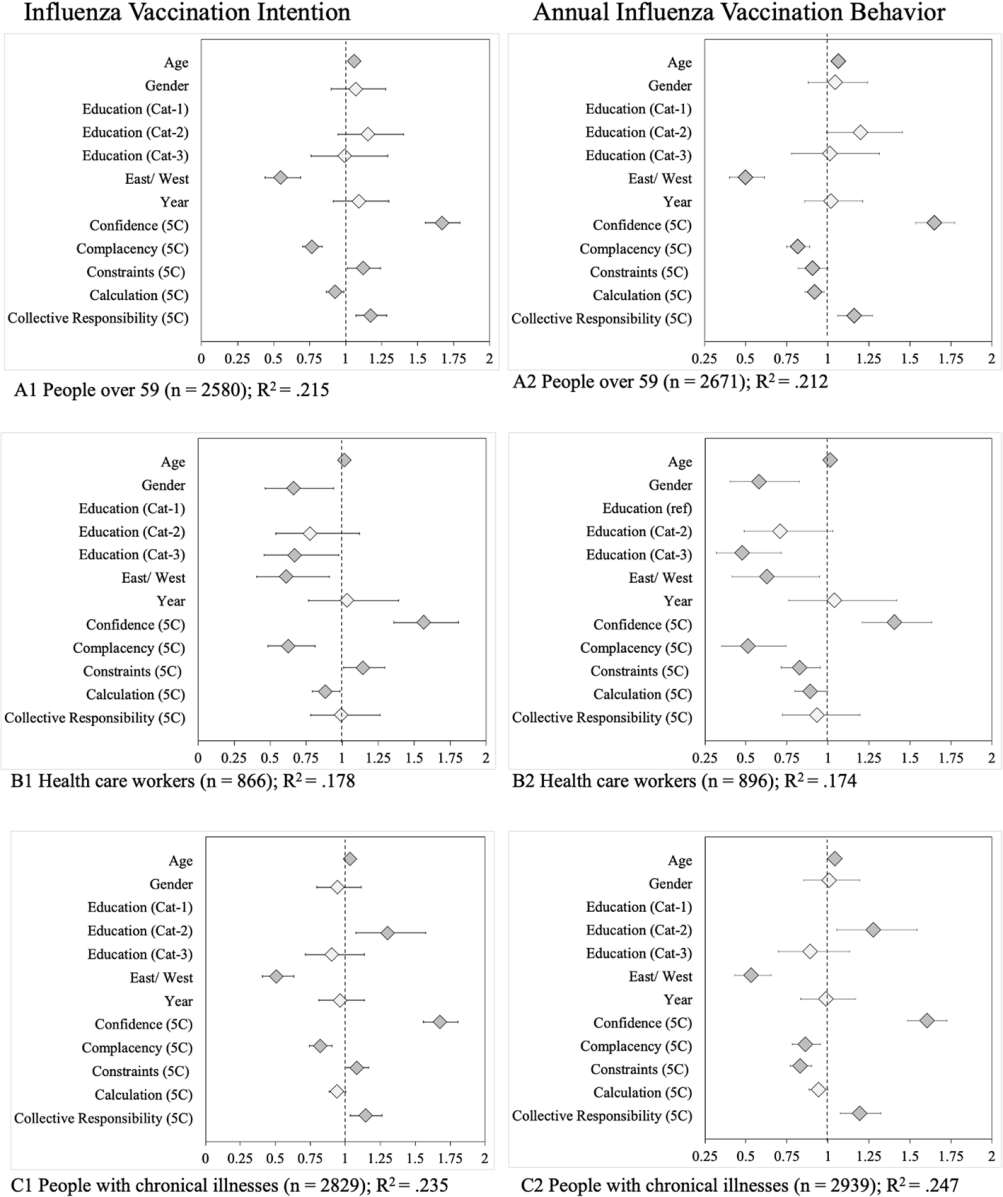
Results of regression analyses on the intention to get the annual influenza vaccination and previous behaviour (‘Do you plan to get vaccinated in the upcoming influenza season?’ and ‘Do you get regular vaccinations against seasonal influenza every year?’). *Note.* Diamonds indicate the odds ratios and whiskers 95% confidence intervals. Dark grey diamonds mark statistically significant predictors (*p* < 0.05). Gender (reference category: male), education (low), East/West (East), and year (2016) are dummy-coded variables

Self-reported annual vaccination behaviour differed between risk groups. Figure 6 shows the analyses for the three influenza risk groups (Panel A: over 59 years of age; B: healthcare workers; C: people with chronic illnesses, where the right panels show previous influenza vaccination behaviour; see also Supplementary Table S5). Among people over 59 years of age, 45% (in 2016, *n* = 764) and 47% (in 2018, *n* = 500) reported having received the vaccine. The self-reported vaccination status of people with chronic illnesses was lower in both 2016 (40.2%, *n* = 656) and 2018 (35.9%, *n* = 516). More annual influenza vaccination behaviour was predicted by older age, East residence, higher Confidence, higher Collective Responsibility (except for healthcare workers), and lower Constraints (except for people over 59). Higher Complacency (lack of risk perception) and higher Constraints (except for people over 59) were related to fewer annual influenza vaccinations. People with chronic illnesses and low education were less likely to regularly receive the seasonal influenza vaccine.

##### Risk groups’ intention to get vaccinated against seasonal influenza

The intention to get vaccinated against seasonal influenza was analysed for the same risk groups (Fig. 6, left panels). Stronger vaccination intentions were associated with higher Confidence, higher Constraints, and higher Collective Responsibility (the latter except for healthcare workers). Weaker vaccination intentions were associated with higher Complacency and, for people over the age of 59 years and healthcare workers, with higher Calculation of risks and benefits. People with chronic illnesses and low education levels were less likely to want the seasonal influenza vaccine.

## Discussion

This study combined data from two representative telephone surveys on the vaccination behaviours of Germans – one conducted in 2016 and another in 2018 [6, 7]. Analyses revealed that the 5C antecedents of vaccination were associated with missed opportunities of vaccination (MOVs), previous vaccination behaviours, and future intentions to vaccinate. In contrast to the demographic variables, the psychological antecedents were significant predictors of different vaccine behaviours in the general sample and within the six target groups. Significant demographic variables for higher vaccination intention and previous vaccination behaviour were higher age (for influenza vaccination) and residing in East Germany. The relevance of East German residence for pro-vaccination behaviour is in line with the results of other studies, especially for target groups like people over the age of 59 years and chronically ill people [20]. This may be due to the historically different vaccination policies in the former West and East Germany regions: In East Germany, the healthcare system was centrally controlled and overseen by the socialist government. Vaccination programmes were compulsory and closely monitored, whereas, in West Germany, vaccinations were recommended, but the decision and responsibility were on the individuals’ side [21]. Due to this difference in systems, various disparities in decision-making structures may have emerged. One possible explanation for the existing differences is the perpetuation of previous behaviour [22, 23]. The data show that vaccination behaviour was relatively stable for both time points (except that influenza vaccination decreased).

Within the six target groups, the 5C are of varying importance in explaining vaccination behaviour. Confidence, i.e. trust in the safety and effectiveness of vaccinations, was crucial across all target groups. In addition to Confidence, Collective Responsibility plays a significant role: individuals aged over 59, who had received vaccinations such as influenza do so not only for their own protection but also to safeguard others through their vaccination. Therefore, it may be advisable to further educate this target group about community protection as long as the vaccine provides herd immunity.

For participants with chronic illnesses, the factor of Complacency permeates the analyses of behaviour and intention. Our study reveals that individuals with chronic conditions who are less willing to be vaccinated also perceive their conditions as less risky. Targeted knowledge campaigns elucidating the connections between chronic illnesses and the risk of severe disease progression could be beneficial in addressing this issue [24]. A second relevant factor in the analyses for chronically ill individuals is Constraints. Perceiving barriers are closely tied to whether they decide in favour of or against vaccination. A broader and more visible vaccination offering by specialized medical practitioners and pharmacies could be helpful in this regard [25].

In our study, people with migratory background represent only a small sample; in addition, the survey was conducted in German, which excluded participants who cannot speak German. People with a migratory background constitute a both under-described and under-represented sample in psychological research [26]. We should translate the insights from our results into first careful recommendations even though our results might be under-describing the target group as well. People with a migration background perceive Constraints, which are related to whether they consciously skip a vaccination or avail themselves of influenza vaccination. We highlight the need to conduct studies within this diverse sample offering multiple languages to understand existing barriers and how they can be structurally dismantled most effectively. Furthermore, it is crucial to ascertain whether the barriers are similarly perceived across all subgroups of individuals with a migratory background or if additional differences can be identified within these groups.

Parents showed a consistent pattern of the 5C antecedents across their own self-reported vaccination behaviours. Confidence and Collective Responsibility can both predict differences between vaccinated and unvaccinated parents. Those 5C antecedents can be addressed with benefit–risk information and [27] educational interventions [28, 29] to increase vaccine acceptance and behaviour. Parents reporting greater Constraints in their daily lives were less likely to having received vaccinations and more likely to have missed vaccination. Remarkably, this predictor was not significant for the influenza vaccine. One potential explanation is that influenza vaccination is often offered at workplaces [30]. Reducing practical barriers, for example in the form of on-site, work-place related vaccinations, can facilitate pro-vaccination behaviour. Even vaccinating parents when they are visiting their child’s doctor may reduce Constraints.

In the case of pregnant women, it is noteworthy that women in East Germany significantly more often declined vaccination compared to their counterparts in western Germany. This deviation contrasts with the pattern observed in the target group of people at the age of 60 or older, where residence in East Germany is associated with higher vaccination intention and behaviour. These findings suggest that the generally positive attitude towards vaccinations may not automatically transfer to subsequent generations, indicating the need for targeted interventions to address this trend in the younger generations in this region.

Lastly, we turn our attention to healthcare professionals. It is noteworthy that within this group, trust in the safety and effectiveness of vaccinations significantly influences the vaccination decision. Moreover, Complacency also differs between the vaccinated and the unvaccinated: the higher the individual perceived risks of vaccine-preventable diseases, the greater the willingness to get vaccinated. Interestingly, Collective Responsibility, indicating the significance of vaccination for others, does not exhibit a significant correlation with uptake or intentions within this group. Therefore, based on this study, it is not apparent that campaigns promoting community protection would promise significant success among healthcare professionals. Instead, efforts should be directed towards informing this target group about the individual risks of vaccine-preventable diseases and thoroughly investigating the specific reasons behind the erosion of trust in vaccinations. Numerous qualitative studies and meta-analyses already shed light on the misinformation landscape within this target group [31–34].

In general, the results help us identify not only general reasons for vaccine hesitancy but also target groups that express special needs. We developed an R-shiny app (bit.ly/VacPattern) which produces figures as displayed in the paper to enable stakeholders to explore the data and identify starting points for interventions. The available samples can be stratified according to risk groups, and analyses can be conducted for different dependent variables. The underlying regressions include age, gender, education, region, and the 5C variables as predictors. The origin of the data is regular German monitoring, and the app will be updated when new data are available.

Future research will show if and how the absolute levels of the determinants (5C) changed over the course of the pandemic as well as their relation to vaccination intentions and behaviours. The present data suggest that if we see changes in general Confidence, this could affect vaccination intention and behaviour for many vaccines. Improvements in access by removing practical barriers (e.g. vaccines are offered in pharmacies and at the workplace, electronic reminder systems are created [35]) could have long-term positive effects. Having experienced the threat that infectious diseases pose to whole societies during the pandemic may affect how people generally perceive infectious diseases and how they evaluate the social benefit of vaccination. Yet, the lack of herd immunity and the respective public discussion about COVID-19 vaccines may have damaged trust in herd immunity [36]. People with a migratory background seem especially vulnerable as access was already an issue before the pandemic. As research from the pandemic shows, people with a migratory background have had, among others, fewer possibilities to isolate, higher risks of infection at work, and fewer chances to obtain evidence-based information that has been targeted or translated [37]. For healthcare workers, trust in the safety and effectiveness of vaccines was already an issue before the pandemic [38]. The decision of some governments (also of the German government) to make vaccination against COVID-19 mandatory for healthcare workers might have affected people’s trust in the system that administers vaccines [39]. This will have to be observed in the future and may be an important consequence of the pandemic.

### Strengths and limitations

This research has some limitations. The 5C antecedents of vaccination were assessed in relation to vaccines in general, even though the 5C scale can also be adapted to specific vaccines. Thus, using the general scale to predict specific vaccination behaviours may have overlooked minimal particularities in the patterns. Previous research has found that for the influenza vaccine, for example, there is a special pattern of the importance of vaccine antecedents [40]. Although we used the general 5C scale and did not include vaccine-specific 5C questions, these patterns were replicated. Future studies should consider asking about the specific behaviour of interest (e.g. is your vaccination status regarding [X] up to date) and adapt the 5C, wherever possible. Next, the drop-out rate of around 51%–62% is comparable with that of other CATI studies [41] but may still indicate sampling bias. Fieldwork studies covering participants across the whole spectrum between denialism and full acceptance of vaccination are highly relevant to validate the results of the present study. Even if the data might not include participants in extreme denial of all vaccines, there is still enough moderate hesitancy in the sample, as the MOV analysis shows. Using a telephone survey rather than a web-based survey was especially valuable for the subgroups of people over the age of 59 and people with chronic illnesses because this approach also reached participants who do not use the internet very often [42]. Nevertheless, especially within the subgroup of people with a migratory background, we do not know whether the effects of a language barrier affected their participation in the study and their access to the health system to the same extent. Further research should attempt to replicate these results with multilingual questionnaires and might also provide further insights into possible barriers to vaccination and healthcare in general. Lastly, the survey time stretched towards the end of the summer holidays in Germany. Even though parents were able to answer in every federal state outside of the school holiday season, the start for the influenza vaccination season is the beginning of October, so we might have asked somewhat early and therefore underestimated the intentions for the influenza vaccination.

The explained variance was low in most of the logistic regressions, even though we applied a group of sociodemographic variables and the 5C as psychological antecedents. The behavioural time frame of five years prior to the study is relatively small considering that most vaccinations for young adults are applied at larger time intervals (6–10 years). For annual influenza vaccination, the explanatory power was higher, which supports this line of thinking as the reported behaviour was shown more frequently.

Given these few but important limitations, the results of the analyses show a pattern of vaccine acceptance in the general population as well as in target groups in Germany before the COVID-19 pandemic. The pre-pandemic results are needed to find any possible secondary pandemic effects regarding other vaccinations. With the intense discussion and the ‘infodemic’ [43] regarding COVID-19 and the respective vaccination from 2020 to 2022, it will be of utmost importance to estimate which groups have changed their feelings and thinking about vaccination and the resulting effects on vaccination behaviour. A study conducted during the pandemic has already shown that parents had their children vaccinated less against childhood diseases, especially when they had lower Confidence in vaccines [44]. This means that barriers that arose during the pandemic may have led people to miss vaccinations. In fact, WHO and UNICEF have already sounded the alarm, as childhood vaccinations went down again after a steady global increase over the last few years [45]. It will be especially interesting to determine whether the phenomenon of higher vaccine uptake in the eastern federal states of Germany will survive the pandemic – as there was less trust in the government during the pandemic and more protests against pandemic measures than in western Germany [46]. Future data could show whether mistrust in authorities and suspicion towards the COVID-19 vaccines will translate into increased general vaccine hesitancy in individual and parental vaccination decisions.

## Conclusion

Embedding the 5C in the regular monitoring of vaccination behaviour will be useful in monitoring the overall status quo and detecting changes in underlying psychological antecedents early on. The results of this study provide crucial information about subgroups and their patterns of vaccine hesitancy. Although Confidence in safety and effectiveness is an important antecedent, so are underestimation of risk, perceived barriers, Collective Responsibility, and the need to calculate risks and benefits. This article serves as a crucial reference point for future research on the impact of the pandemic on vaccination behaviours.

### Supplementary Information

Supplementary Material 1: Sample Demographics.

Supplementary Material 2: Tables for Regression analyses.

## Data Availability

Raw data, materials and analysis protocols can be found on the Open Science Framework (OSF: https://osf.io/ezg2k/?view_only=58be5993ecda485baf803d1180803560).
