# Supplementary material for: Exploring pre-pandemic patterns of vaccine decision-making with the 5C model: results from representative surveys in 2016 and 2018
Source: BMC Public Health. 2024 Apr 30;24:1205. doi: 10.1186/s12889-024-18674-9 (PMC11061918; doi:10.1186/s12889-024-18674-9)
Supplement: Supplementary file 1 — Supplementary Material 1: Sample Demographics. [file 12889_2024_18674_MOESM1_ESM.docx]

**Table S1.** Demographics and Subgroup Distributions.

|  | |  | Total | 2016 | 2018 | Census 2011 |
| --- | --- | --- | --- | --- | --- | --- |
| Subgroup distribution | | | N = 10,066 | N=5,012 | N=5,054 |  |
|  | Age group >60 | Under 60 | 7136 (71.1%) | 3209 (64%) | 3927 (77.7%) | 73% |
|  |  | 60+ | 2907 (28.9%) | 1790 (35.7%) | 1117 (22.1%) | 27% |
|  | Working in health care | Yes | 956 (14.5%) | 456 (15.4%) | 500 (13.7%) | 6.3% |
|  |  | No | 5657 (85.5%) | 2503 (84.6%) | 3154 (86.3%) | 93.7% |
|  |  | Missing data | 3453 | 2053 | 1400 |  |
|  | Parents | Yes, at least one child | 2152 (21.4%) | 1092 (21.8%) | 1060 (21.0%) | 32.7% |
|  |  | No | 7914 (78.6%) | 3920 (78.2%) | 3994 (79.0%) | 67.3% |
|  | Pregnant women | Yes, currently pregnant | 1003 (33.1%) | 502 (35.3%) | 501 (31.2%) | 2.8% |
|  |  | No | 2027 (66.9%) | 922 (64.7%) | 1105 (68.8%) | 97.2% |
|  |  | Missing data | 7036 | 3588 | 3448 |  |
|  | Chronical illness | Yes | 3223 (32.1%) | 1711 (34.1%) | 1512 (29.9%) |  |
|  |  | No | 6811 (67.9%) | 3284 (65.5%) | 3527 (69.8%) |  |
|  | Migratory background | Yes | 719 (7.1%) | 352 (7%) | 367 (7.3%) | 13.1% |
|  |  | No | 9347 (92.9%) | 4460 (93%) | 4687 (92.7%) | 86.9% |
| Demographic distribution | | |  |  |  |  |
|  | Age |  | *M* = 48.34 *SD* = 17.8 | *M* = 50.74 *SD* = 18.1 | *M* = 45.96 *SD* = 17.1 |  |
|  | Gender | female | 5949 (59.1%) | 3055 (61.0%) | 2894 (57.3%) | 52% |
|  |  | male | 4117 (40.9%) | 1957 (39.0%) | 2160 (42.7%) | 48% |
|  | Education | Low | 3757 (39%) | 2021 (42.3%) | 1736 (35.7%) | 41.1% |
|  |  | Medium | 2493 (25.9%) | 1169 (24.4%) | 1324 (27.3%) | 28.9% |
|  |  | High | 3388 (35.2%) | 1592 (33.3%) | 1796 (37.0%) | 26.6% |
|  | East/west heritage | East | 1514 (15.8%) | 816 (17.1%) | 698 (14.5%) | 19.8% |
|  |  | West | 8059 (84.2%) | 3957 (82.9%) | 4102 (85.5%) | 80.2% |

*Note*. Distribution of demographic variables over the two survey samples (2016, 2018) and for the complete analyses sample of the two consecutive surveys. Systematic missings due to filtered questions are displayed where needed. Differences to the single analyses might occur due to missing data on the dependent variables. Due to these missings, we do not include sample weighting variables that help to reach representativeness. Deviances from representative distributions can be seen in the last Column, where census data from 2011 are displayed. The samples are slightly higher educated and parents and pregnant women are higher represented compared to the German population.
