# Supplementary material for: Exploring pre-pandemic patterns of vaccine decision-making with the 5C model: results from representative surveys in 2016 and 2018
Source: BMC Public Health. 2024 Apr 30;24:1205. doi: 10.1186/s12889-024-18674-9 (PMC11061918; doi:10.1186/s12889-024-18674-9)

Supplement B: Logistic Regressions supplementing the Figures 1-4

**Table S2:** *Logistic Regressions for missed opportunities of vaccination: “Did it happen at least once during the last five years that you chose not to get vaccinated?” in the Full Sample and all subgroups.*

|  | **Full Sample** | | | **(A) People over 60** | | | **(B) Chronical illnesses** | | | **(C) Migratory background** | | | **(D) HCW** | | | **(E) Parents** | | | **(F) Pregnant women** | | |
| --- | --- | --- | --- | --- | --- | --- | --- | --- | --- | --- | --- | --- | --- | --- | --- | --- | --- | --- | --- | --- | --- |
| *Predictors* | *OR* | *CI* | *p* | *OR* | *CI* | *p* | *OR* | *CI* | *p* | *OR* | *CI* | *p* | *OR* | *CI* | *p* | *OR* | *CI* | *p* | *OR* | *CI* | *p* |
| Age | 1.00 | 1.00 – 1.00 | 0.528 | 0.99 | 0.98 – 1.00 | 0.094 | 1.00 | 0.99 – 1.00 | 0.756 | 1.00 | 0.99 – 1.01 | 0.849 | 1.01 | 1.00 – 1.03 | **0.023** | 1.00 | 0.99 – 1.02 | 0.602 | 0.99 | 0.95 – 1.02 | 0.421 |
| Gender | 0.98 | 0.88 – 1.08 | 0.622 | 0.92 | 0.76 – 1.10 | 0.351 | 1.01 | 0.86 – 1.20 | 0.868 | 1.11 | 0.74 – 1.67 | 0.623 | 1.02 | 0.71 – 1.47 | 0.915 | 1.08 | 0.86 – 1.36 | 0.512 |  |  |  |
| Education (medium) | 1.09 | 0.97 – 1.24 | 0.159 | 1.20 | 0.92 – 1.54 | 0.172 | 1.02 | 0.82 – 1.28 | 0.849 | 1.44 | 0.89 – 2.35 | 0.139 | 1.29 | 0.90 – 1.86 | 0.164 | 1.08 | 0.82 – 1.41 | 0.584 | 0.97 | 0.64 – 1.46 | 0.869 |
| Education (high) | 1.12 | 1.00 – 1.26 | **0.047** | 1.11 | 0.91 – 1.36 | 0.306 | 1.09 | 0.90 – 1.33 | 0.373 | 0.95 | 0.60 – 1.51 | 0.842 | 1.11 | 0.76 – 1.61 | 0.594 | 1.13 | 0.88 – 1.44 | 0.345 | 0.95 | 0.66 – 1.39 | 0.808 |
| East/ West | 1.10 | 0.96 – 1.26 | 0.165 | 1.38 | 1.09 – 1.76 | **0.007** | 1.19 | 0.96 – 1.50 | 0.123 | 0.75 | 0.34 – 1.79 | 0.494 | 1.25 | 0.82 – 1.94 | 0.319 | 0.96 | 0.72 – 1.27 | 0.760 | 0.62 | 0.40 – 0.95 | **0.026** |
| Survey 2018 (vs. 2016) | 0.94 | 0.86 – 1.04 | 0.254 | 0.94 | 0.78 – 1.13 | 0.485 | 1.06 | 0.90 – 1.26 | 0.482 | 0.80 | 0.54 – 1.18 | 0.264 | 1.13 | 0.83 – 1.52 | 0.436 | 0.88 | 0.71 – 1.07 | 0.202 | 1.02 | 0.76 – 1.38 | 0.895 |
| Confidence (5C) | 0.80 | 0.77 – 0.83 | **<0.001** | 0.85 | 0.79 – 0.91 | **<0.001** | 0.84 | 0.78 – 0.90 | **<0.001** | 0.88 | 0.76 – 1.02 | 0.079 | 0.77 | 0.68 – 0.88 | **<0.001** | 0.75 | 0.68 – 0.82 | **<0.001** | 0.70 | 0.61 – 0.80 | **<0.001** |
| Constraints (5C) | 1.34 | 1.29 – 1.39 | **<0.001** | 1.17 | 1.06 – 1.29 | **0.001** | 1.30 | 1.22 – 1.40 | **<0.001** | 1.35 | 1.17 – 1.55 | **<0.001** | 1.29 | 1.15 – 1.45 | **<0.001** | 1.36 | 1.27 – 1.46 | **<0.001** | 1.36 | 1.21 – 1.52 | **<0.001** |
| Coll. Respon-sibility (5C) | 0.91 | 0.86 – 0.97 | **0.002** | 0.94 | 0.86 – 1.02 | 0.146 | 0.94 | 0.86 – 1.04 | 0.211 | 1.09 | 0.90 – 1.33 | 0.410 | 0.89 | 0.72 – 1.11 | 0.296 | 0.86 | 0.75 – 0.98 | **0.026** | 0.93 | 0.73 – 1.20 | 0.575 |
| Calculation (5C) | 0.99 | 0.96 – 1.03 | 0.665 | 1.00 | 0.94 – 1.07 | 0.978 | 1.01 | 0.95 – 1.08 | 0.691 | 0.95 | 0.83 – 1.08 | 0.431 | 1.01 | 0.90 – 1.13 | 0.919 | 1.00 | 0.93 – 1.08 | 0.907 | 1.05 | 0.93 – 1.18 | 0.449 |
| Complacency (5C) | 1.08 | 1.03 – 1.14 | **0.003** | 1.07 | 0.98 – 1.17 | 0.116 | 1.09 | 0.99 – 1.19 | 0.062 | 1.10 | 0.92 – 1.30 | 0.286 | 0.98 | 0.81 – 1.18 | 0.864 | 1.05 | 0.92 – 1.19 | 0.492 | 0.88 | 0.70 – 1.08 | 0.226 |
| N | 8892 | | | 2646 | | | 2910 | | | 617 | | | 892 | | | 1990 | | | 925 | | |
| R^2^ Tjur | 0.050 | | | 0.024 | | | 0.039 | | | 0.051 | | | 0.050 | | | 0.066 | | | 0.071 | | |

**Table S3:** *Logistic Regressions for vaccination demand: “Did you receive a vaccination in the last five years (since the summer of 2011/2013)? in the Full Sample and all subgroups.*

|  | **Full Sample** | | | **(A) People over 60** | | | **(B) Chronical illnesses** | | | **(C) Migratory background** | | | **(D) HCW** | | | **(E) Parents** | | | **(F) Pregnant women** | | |
| --- | --- | --- | --- | --- | --- | --- | --- | --- | --- | --- | --- | --- | --- | --- | --- | --- | --- | --- | --- | --- | --- |
| *Predictors* | *OR* | *CI* | *p* | *OR* | *CI* | *p* | *OR* | *CI* | *p* | *OR* | *CI* | *p* | *OR* | *CI* | *p* | *OR* | *CI* | *p* | *OR* | *CI* | *p* |
| Age | 0.99 | 0.99 – 0.99 | **<0.001** | 1.00 | 0.99 – 1.02 | 0.769 | 0.99 | 0.98 – 1.00 | **0.002** | 0.99 | 0.98 – 1.00 | **0.032** | 0.98 | 0.97 – 0.99 | **0.006** | 0.99 | 0.97 – 1.01 | 0.258 | 1.02 | 0.98 – 1.05 | 0.377 |
| Gender | 1.03 | 0.93 – 1.14 | 0.582 | 1.07 | 0.89 – 1.29 | 0.478 | 1.06 | 0.88 – 1.27 | 0.544 | 0.95 | 0.65 – 1.37 | 0.768 | 0.81 | 0.52 – 1.25 | 0.361 | 0.71 | 0.55 – 0.90 | **0.005** |  |  |  |
| Education (medium) | 0.91 | 0.80 – 1.04 | 0.167 | 0.91 | 0.70 – 1.19 | 0.502 | 0.75 | 0.59 – 0.94 | **0.015** | 0.84 | 0.53 – 1.32 | 0.452 | 0.75 | 0.49 – 1.13 | 0.171 | 0.78 | 0.59 – 1.03 | 0.080 | 0.96 | 0.64 – 1.46 | 0.863 |
| Education (high) | 0.99 | 0.88 – 1.12 | 0.887 | 0.94 | 0.77 – 1.16 | 0.579 | 0.94 | 0.76 – 1.17 | 0.587 | 1.32 | 0.87 – 2.01 | 0.190 | 1.04 | 0.66 – 1.63 | 0.873 | 0.98 | 0.76 – 1.27 | 0.876 | 1.42 | 0.96 – 2.09 | 0.080 |
| East/ West | 0.60 | 0.51 – 0.70 | **<0.001** | 0.47 | 0.36 – 0.60 | **<0.001** | 0.48 | 0.37 – 0.63 | **<0.001** | 0.54 | 0.21 – 1.21 | 0.153 | 0.58 | 0.33 – 0.98 | 0.051 | 0.67 | 0.48 – 0.92 | **0.015** | 0.66 | 0.39 – 1.06 | 0.097 |
| Survey 2018 (vs. 2016) | 0.97 | 0.87 – 1.07 | 0.496 | 1.03 | 0.85 – 1.24 | 0.793 | 0.90 | 0.75 – 1.09 | 0.282 | 0.72 | 0.50 – 1.03 | 0.071 | 1.36 | 0.95 – 1.93 | 0.090 | 0.91 | 0.74 – 1.13 | 0.388 | 0.79 | 0.58 – 1.08 | 0.145 |
| Confidence (5C) | 1.43 | 1.38 – 1.49 | **<0.001** | 1.47 | 1.37 – 1.58 | **<0.001** | 1.35 | 1.25 – 1.45 | **<0.001** | 1.39 | 1.21 – 1.59 | **<0.001** | 1.44 | 1.25 – 1.66 | **<0.001** | 1.40 | 1.28 – 1.53 | **<0.001** | 1.50 | 1.31 – 1.73 | **<0.001** |
| Constraints (5C) | 0.83 | 0.80 – 0.86 | **<0.001** | 0.93 | 0.84 – 1.03 | 0.141 | 0.84 | 0.78 – 0.91 | **<0.001** | 0.87 | 0.76 – 1.00 | 0.054 | 0.78 | 0.68 – 0.89 | **<0.001** | 0.80 | 0.74 – 0.87 | **<0.001** | 0.76 | 0.68 – 0.85 | **<0.001** |
| Coll. Respon-sibility (5C) | 1.23 | 1.16 – 1.30 | **<0.001** | 1.22 | 1.11 – 1.33 | **<0.001** | 1.26 | 1.15 – 1.39 | **<0.001** | 1.15 | 0.97 – 1.36 | 0.114 | 1.19 | 0.93 – 1.49 | 0.151 | 1.23 | 1.07 – 1.41 | **0.003** | 1.34 | 1.05 – 1.71 | **0.019** |
| Calculation (5C) | 0.97 | 0.94 – 1.01 | 0.186 | 1.03 | 0.96 – 1.10 | 0.415 | 0.99 | 0.92 – 1.06 | 0.717 | 1.04 | 0.92 – 1.17 | 0.557 | 0.84 | 0.72 – 0.97 | **0.017** | 0.92 | 0.85 – 1.00 | **0.042** | 0.89 | 0.78 – 1.01 | 0.064 |
| Complacency (5C) | 0.83 | 0.78 – 0.87 | **<0.001** | 0.81 | 0.74 – 0.88 | **<0.001** | 0.85 | 0.78 – 0.94 | **0.001** | 0.76 | 0.65 – 0.89 | **0.001** | 0.71 | 0.58 – 0.85 | **<0.001** | 0.86 | 0.76 – 0.99 | **0.030** | 0.99 | 0.81 – 1.23 | 0.934 |
| N | 8767 | | | 2623 | | | 2873 | | | 606 | | | 884 | | | 1952 | | | 901 | | |
| R^2^ Tjur | 0.087 | | | 0.101 | | | 0.075 | | | 0.101 | | | 0.117 | | | 0.078 | | | 0.108 | | |

**Table S4:** *Logistic Regressions for influenza vaccination behavior: “Which of the following diseases have you been vaccinated against in the last five years? [Influenza]” in the Full Sample and all subgroups.*

|  | **Full Sample** | | | **(A) People over 60** | | | **(B) Chronical illnesses** | | | **(C) Migratory background** | | | **(D) HCW** | | | **(E) Parents** | | | **(F) Pregnant women** | | |
| --- | --- | --- | --- | --- | --- | --- | --- | --- | --- | --- | --- | --- | --- | --- | --- | --- | --- | --- | --- | --- | --- |
| *Predictors* | *OR* | *CI* | *p* | *OR* | *CI* | *p* | *OR* | *CI* | *p* | *OR* | *CI* | *p* | *OR* | *CI* | *p* | *OR* | *CI* | *p* | *OR* | *CI* | *p* |
| Age | 1.04 | 1.04 – 1.05 | **<0.001** | 1.09 | 1.07 – 1.11 | **<0.001** | 1.04 | 1.03 – 1.05 | **<0.001** | 1.05 | 1.03 – 1.06 | **<0.001** | 1.01 | 1.00 – 1.02 | 0.112 | 1.02 | 1.00 – 1.04 | **0.047** | 1.07 | 1.02 – 1.11 | **0.002** |
| Gender | 0.95 | 0.85 – 1.06 | 0.339 | 1.00 | 0.80 – 1.24 | 0.979 | 0.93 | 0.77 – 1.13 | 0.469 | 0.79 | 0.50 – 1.27 | 0.334 | 0.64 | 0.44 – 0.94 | **0.021** | 0.82 | 0.64 – 1.05 | 0.115 |  |  |  |
| Education (medium) | 0.84 | 0.73 – 0.97 | **0.017** | 0.87 | 0.64 – 1.19 | 0.390 | 0.86 | 0.67 – 1.10 | 0.234 | 0.71 | 0.38 – 1.30 | 0.264 | 0.75 | 0.51 – 1.10 | 0.139 | 0.93 | 0.68 – 1.25 | 0.619 | 0.59 | 0.36 – 0.96 | **0.034** |
| Education (high) | 0.99 | 0.88 – 1.13 | 0.933 | 1.01 | 0.79 – 1.29 | 0.942 | 0.96 | 0.78 – 1.20 | 0.744 | 0.99 | 0.59 – 1.65 | 0.957 | 1.46 | 1.00 – 2.15 | 0.053 | 1.40 | 1.08 – 1.83 | **0.012** | 1.20 | 0.79 – 1.82 | 0.399 |
| East/ West | 0.56 | 0.49 – 0.64 | **<0.001** | 0.61 | 0.46 – 0.79 | **<0.001** | 0.59 | 0.46 – 0.75 | **<0.001** | 0.59 | 0.21 – 1.55 | 0.288 | 0.57 | 0.37 – 0.88 | **0.011** | 0.56 | 0.42 – 0.76 | **<0.001** | 0.43 | 0.27 – 0.69 | **0.001** |
| Survey 2018 (vs. 2016) | 0.90 | 0.81 – 1.00 | 0.052 | 0.90 | 0.72 – 1.11 | 0.323 | 0.86 | 0.71 – 1.05 | 0.134 | 0.97 | 0.62 – 1.54 | 0.911 | 0.99 | 0.72 – 1.35 | 0.939 | 0.86 | 0.69 – 1.07 | 0.168 | 1.01 | 0.72 – 1.41 | 0.957 |
| Confidence (5C) | 1.35 | 1.28 – 1.42 | **<0.001** | 1.43 | 1.31 – 1.56 | **<0.001** | 1.36 | 1.25 – 1.47 | **<0.001** | 1.30 | 1.06 – 1.59 | **0.012** | 1.35 | 1.16 – 1.57 | **<0.001** | 1.38 | 1.23 – 1.55 | **<0.001** | 1.26 | 1.05 – 1.52 | **0.014** |
| Constraints (5C) | 1.01 | 0.97 – 1.06 | 0.578 | 1.02 | 0.90 – 1.16 | 0.791 | 1.01 | 0.93 – 1.10 | 0.769 | 1.22 | 1.01 – 1.47 | **0.035** | 1.06 | 0.92 – 1.22 | 0.414 | 0.96 | 0.88 – 1.05 | 0.373 | 1.03 | 0.89 – 1.20 | 0.670 |
| Coll. Respon-sibility (5C) | 1.05 | 0.97 – 1.13 | 0.243 | 0.95 | 0.83 – 1.07 | 0.389 | 1.00 | 0.88 – 1.14 | 0.940 | 1.07 | 0.84 – 1.38 | 0.582 | 0.92 | 0.72 – 1.19 | 0.521 | 1.17 | 0.97 – 1.45 | 0.117 | 1.93 | 1.18 – 3.73 | **0.022** |
| Calculation (5C) | 0.92 | 0.88 – 0.96 | **<0.001** | 0.87 | 0.80 – 0.95 | **0.002** | 0.91 | 0.85 – 0.98 | **0.011** | 1.00 | 0.85 – 1.18 | 0.985 | 0.91 | 0.81 – 1.02 | 0.098 | 0.97 | 0.90 – 1.05 | 0.506 | 0.97 | 0.86 – 1.09 | 0.582 |
| Complacency (5C) | 0.89 | 0.83 – 0.95 | **0.001** | 0.83 | 0.74 – 0.93 | **0.001** | 0.85 | 0.76 – 0.95 | **0.004** | 0.99 | 0.77 – 1.26 | 0.912 | 0.75 | 0.57 – 0.96 | **0.030** | 0.88 | 0.73 – 1.05 | 0.176 | 0.98 | 0.73 – 1.31 | 0.914 |
| N | 6458 | | | 1895 | | | 2174 | | | 387 | | | 698 | | | 1429 | | | 647 | | |
| R^2^ Tjur | 0.147 | | | 0.112 | | | 0.143 | | | 0.176 | | | 0.091 | | | 0.057 | | | 0.076 | | |

**Table S5:** *Logistic Regressions for Annual influenza vaccination. “Do you get regular vaccinations against the seasonal influenza every year?”*

|  | **(A1) People over 60** | | | **(B1) HCW** | | | **(C1) Chronical illnesses** | | |
| --- | --- | --- | --- | --- | --- | --- | --- | --- | --- |
| *Predictors* | *OR* | *CI* | *p* | *OR* | *CI* | *p* | *OR* | *CI* | *p* |
| Age | 1.06 | 1.05 – 1.08 | **<0.001** | 1.01 | 1.00 – 1.03 | **0.043** | 1.04 | 1.03 – 1.05 | **<0.001** |
| Gender | 1.05 | 0.88 – 1.24 | 0.614 | 0.58 | 0.40 – 0.83 | **0.003** | 1.01 | 0.85 – 1.19 | 0.932 |
| Education (medium) | 0.84 | 0.66 – 1.08 | 0.182 | 0.67 | 0.45 – 1.00 | 0.053 | 0.70 | 0.55 – 0.87 | **0.002** |
| Education (high) | 0.83 | 0.69 – 1.01 | 0.063 | 1.41 | 0.97 – 2.05 | 0.071 | 0.78 | 0.65 – 0.95 | **0.012** |
| East/ West | 0.50 | 0.40 – 0.61 | **<0.001** | 0.63 | 0.42 – 0.95 | **0.027** | 0.53 | 0.43 – 0.65 | **<0.001** |
| Survey 2018 (vs. 2016) | 1.02 | 0.86 – 1.21 | 0.820 | 1.04 | 0.76 – 1.42 | 0.813 | 0.99 | 0.83 – 1.17 | 0.876 |
| Confidence (5C) | 1.65 | 1.54 – 1.77 | **<0.001** | 1.41 | 1.21 – 1.64 | **<0.001** | 1.60 | 1.49 – 1.73 | **<0.001** |
| Constraints (5C) | 0.91 | 0.82 – 1.00 | 0.052 | 0.83 | 0.71 – 0.95 | **0.009** | 0.83 | 0.77 – 0.90 | **<0.001** |
| Coll. Responsibility (5C) | 1.16 | 1.06 – 1.27 | **0.001** | 0.93 | 0.73 – 1.21 | 0.577 | 1.19 | 1.07 – 1.32 | **0.001** |
| Calculation (5C) | 0.92 | 0.86 – 0.98 | **0.007** | 0.89 | 0.80 – 1.00 | **0.045** | 0.94 | 0.88 – 1.00 | 0.055 |
| Complacency (5C) | 0.82 | 0.75 – 0.89 | **<0.001** | 0.51 | 0.34 – 0.71 | **<0.001** | 0.86 | 0.78 – 0.95 | **0.003** |
| N | 2671 | | | 896 | | | 2939 | | |
| R^2^ Tjur | 0.161 | | | 0.124 | | | 0.189 | | |

**Table S6:** *Logistic Regressions for influenza vaccination intention:”Do you plan to get vaccinated in the upcoming influenza season?”.*

|  | **(A1) People over 60** | | | **(B1) HCW** | | | **(C1) Chronical illnesses** | | |
| --- | --- | --- | --- | --- | --- | --- | --- | --- | --- |
| *Predictors* | *OR* | *CI* | *p* | *OR* | *CI* | *p* | *OR* | *CI* | *p* |
| Age | 1.06 | 1.04 – 1.07 | **<0.001** | 1.01 | 1.00 – 1.03 | **0.034** | 1.04 | 1.03 – 1.04 | **<0.001** |
| Gender | 1.07 | 0.90 – 1.28 | 0.446 | 0.66 | 0.47 – 0.94 | **0.022** | 0.94 | 0.80 – 1.11 | 0.489 |
| Education (medium) | 0.86 | 0.67 – 1.11 | 0.238 | 0.86 | 0.60 – 1.24 | 0.422 | 0.69 | 0.56 – 0.86 | **0.001** |
| Education (high) | 0.87 | 0.71 – 1.05 | 0.152 | 1.29 | 0.89 – 1.85 | 0.174 | 0.77 | 0.63 – 0.93 | **0.006** |
| East/ West | 0.55 | 0.44 – 0.69 | **<0.001** | 0.61 | 0.41 – 0.91 | **0.015** | 0.51 | 0.41 – 0.63 | **<0.001** |
| Survey 2018 (vs. 2016) | 1.09 | 0.92 – 1.30 | 0.333 | 1.03 | 0.77 – 1.39 | 0.828 | 0.96 | 0.81 – 1.14 | 0.645 |
| Confidence (5C) | 1.67 | 1.55 – 1.79 | **<0.001** | 1.57 | 1.36 – 1.81 | **<0.001** | 1.68 | 1.56 – 1.81 | **<0.001** |
| Constraints (5C) | 1.12 | 1.01 – 1.24 | **0.028** | 1.14 | 1.01 – 1.30 | **0.033** | 1.08 | 1.01 – 1.17 | **0.030** |
| Coll. Responsibility (5C) | 1.17 | 1.07 – 1.29 | **<0.001** | 0.99 | 0.78 – 1.27 | 0.964 | 1.14 | 1.04 – 1.27 | **0.008** |
| Calculation (5C) | 0.93 | 0.87 – 0.99 | **0.021** | 0.88 | 0.79 – 0.99 | **0.026** | 0.94 | 0.88 – 1.00 | 0.056 |
| Complacency (5C) | 0.77 | 0.70 – 0.84 | **<0.001** | 0.63 | 0.47 – 0.80 | **<0.001** | 0.82 | 0.74 – 0.90 | **<0.001** |
| N | 2580 | | | 866 | | | 2829 | | |
| R^2^ Tjur | 0.164 | | | 0.131 | | | 0.179 | | |

**Correlational tables for the analyses**

**Table S7:** *Means, standard deviations, and correlations with confidence intervals for missed opportunities of vaccination: “Did it happen at least once during the last five years that you chose not to get vaccinated?” in the Full Sample*

| Variable | *M* | *SD* | 1 | 2 | 3 | 4 | 5 | 6 | 7 |
| --- | --- | --- | --- | --- | --- | --- | --- | --- | --- |
|  |  |  |  |  |  |  |  |  |  |
| 1. MOV | 0.27 | 0.45 |  |  |  |  |  |  |  |
|  |  |  |  |  |  |  |  |  |  |
| 2. Age | 49.37 | 16.94 | -.02* |  |  |  |  |  |  |
|  |  |  | [-.04, -.00] |  |  |  |  |  |  |
| 3. Year | 1.51 | 0.50 | -.02 | -.15** |  |  |  |  |  |
|  |  |  | [-.04, .01] | [-.17, -.12] |  |  |  |  |  |
|  |  |  |  |  |  |  |  |  |  |
| 4. Confidence (5C) | 3.69 | 1.23 | -.13** | -.01 | .03** |  |  |  |  |
|  |  |  | [-.15, -.11] | [-.03, .01] | [.01, .06] |  |  |  |  |
|  |  |  |  |  |  |  |  |  |  |
| 5. Constraints (5C) | 1.62 | 1.19 | .17** | -.19** | .03* | -.00 |  |  |  |
|  |  |  | [.15, .19] | [-.21, -.17] | [.00, .05] | [-.02, .02] |  |  |  |
|  |  |  |  |  |  |  |  |  |  |
| 6. Coll. Responsibility (5C) | 4.69 | 0.83 | -.06** | -.07** | .06** | .13** | -.03** |  |  |
|  |  |  | [-.08, -.04] | [-.09, -.04] | [.04, .08] | [.11, .15] | [-.05, -.01] |  |  |
|  |  |  |  |  |  |  |  |  |  |
| 7. Calculation (5C) | 3.94 | 1.37 | .01 | .11** | -.04** | -.16** | -.04** | -.04** |  |
|  |  |  | [-.01, .03] | [.09, .13] | [-.06, -.01] | [-.18, -.13] | [-.06, -.02] | [-.06, -.02] |  |
|  |  |  |  |  |  |  |  |  |  |
| 8. Complacency (5C) | 1.37 | 0.89 | .08** | .05** | -.05** | -.20** | .07** | -.24** | .03** |
|  |  |  | [.06, .10] | [.03, .08] | [-.07, -.03] | [-.22, -.18] | [.05, .09] | [-.26, -.22] | [.01, .05] |
|  |  |  |  |  |  |  |  |  |  |

*Note.* *M* and *SD* are used to represent mean and standard deviation, respectively. Values in square brackets indicate the 95% confidence interval for each correlation. The confidence interval is a plausible range of population correlations that could have caused the sample correlation (Cumming, 2014). * indicates *p* < .05. ** indicates *p* < .01. MOV = missed opportunities of vaccination.

**Table S8**: *Means, standard deviations, and correlations with confidence intervals for vaccination demand: “Did you receive a vaccination in the last five years (since the summer of 2011/2013)? in the Full Sample and all subgroups.*

| Variable | *M* | *SD* | 1 | 2 | 3 | 4 | 5 | 6 | 7 |
| --- | --- | --- | --- | --- | --- | --- | --- | --- | --- |
|  |  |  |  |  |  |  |  |  |  |
| 1. Demand | 0.74 | 0.44 |  |  |  |  |  |  |  |
|  |  |  |  |  |  |  |  |  |  |
| 2. Age | 49.47 | 16.94 | -.06** |  |  |  |  |  |  |
|  |  |  | [-.08, -.04] |  |  |  |  |  |  |
|  |  |  |  |  |  |  |  |  |  |
| 3. Year | 1.50 | 0.50 | .01 | -.15** |  |  |  |  |  |
|  |  |  | [-.01, .04] | [-.17, -.12] |  |  |  |  |  |
|  |  |  |  |  |  |  |  |  |  |
| 4. Confidence (5C) | 3.69 | 1.23 | .23** | -.01 | .03** |  |  |  |  |
|  |  |  | [.21, .25] | [-.03, .01] | [.01, .06] |  |  |  |  |
|  |  |  |  |  |  |  |  |  |  |
| 5. Constraints (5C) | 1.62 | 1.19 | -.09** | -.19** | .03** | -.01 |  |  |  |
|  |  |  | [-.11, -.07] | [-.21, -.17] | [.01, .05] | [-.03, .02] |  |  |  |
|  |  |  |  |  |  |  |  |  |  |
| 6. Coll. Responsibility (5C) | 4.70 | 0.83 | .13** | -.07** | .06** | .13** | -.03** |  |  |
|  |  |  | [.11, .15] | [-.09, -.05] | [.04, .08] | [.11, .15] | [-.06, -.01] |  |  |
|  |  |  |  |  |  |  |  |  |  |
| 7. Calculation (5C) | 3.94 | 1.36 | -.05** | .11** | -.03** | -.15** | -.04** | -.04** |  |
|  |  |  | [-.07, -.03] | [.09, .13] | [-.05, -.01] | [-.17, -.13] | [-.06, -.01] | [-.06, -.02] |  |
|  |  |  |  |  |  |  |  |  |  |
| 8. Complacency (5C) | 1.38 | 0.90 | -.15** | .06** | -.05** | -.20** | .07** | -.24** | .03** |
|  |  |  | [-.17, -.13] | [.04, .08] | [-.07, -.03] | [-.22, -.18] | [.05, .09] | [-.25, -.22] | [.01, .05] |
|  |  |  |  |  |  |  |  |  |  |

*Note.* *M* and *SD* are used to represent mean and standard deviation, respectively. Values in square brackets indicate the 95% confidence interval for each correlation. The confidence interval is a plausible range of population correlations that could have caused the sample correlation (Cumming, 2014). * indicates *p* < .05. ** indicates *p* < .01.

**Table S9**: *Means, standard deviations, and correlations with confidence intervals for influenza vaccination behavior: “Which of the following diseases have you been vaccinated against in the last five years? [Influenza]” in the Full Sample*

| Variable | *M* | *SD* | 1 | 2 | 3 | 4 | 5 | 6 | 7 |
| --- | --- | --- | --- | --- | --- | --- | --- | --- | --- |
|  |  |  |  |  |  |  |  |  |  |
| 1. Influenza Vaccination | 0.46 | 0.50 |  |  |  |  |  |  |  |
|  |  |  |  |  |  |  |  |  |  |
| 2. Age | 48.32 | 17.76 | .32** |  |  |  |  |  |  |
|  |  |  | [.30, .34] |  |  |  |  |  |  |
|  |  |  |  |  |  |  |  |  |  |
| 3. Year | 1.50 | 0.50 | -.06** | -.13** |  |  |  |  |  |
|  |  |  | [-.08, -.04] | [-.15, -.11] |  |  |  |  |  |
|  |  |  |  |  |  |  |  |  |  |
| 4. Confidence (5C) | 3.67 | 1.24 | .18** | -.00 | .04** |  |  |  |  |
|  |  |  | [.15, .20] | [-.02, .02] | [.02, .06] |  |  |  |  |
|  |  |  |  |  |  |  |  |  |  |
| 5. Constraints (5C) | 1.62 | 1.19 | -.06** | -.19** | .02* | -.00 |  |  |  |
|  |  |  | [-.08, -.04] | [-.21, -.17] | [.00, .04] | [-.02, .02] |  |  |  |
|  |  |  |  |  |  |  |  |  |  |
| 6. Coll. Responsibility (5C) | 4.68 | 0.84 | .02 | -.05** | .06** | .14** | -.03** |  |  |
|  |  |  | [-.00, .05] | [-.07, -.03] | [.04, .08] | [.12, .16] | [-.05, -.01] |  |  |
|  |  |  |  |  |  |  |  |  |  |
| 7. Calculation (5C) | 3.91 | 1.37 | -.04** | .12** | -.03** | -.15** | -.03** | -.04** |  |
|  |  |  | [-.06, -.01] | [.10, .14] | [-.05, -.01] | [-.17, -.13] | [-.05, -.01] | [-.06, -.02] |  |
|  |  |  |  |  |  |  |  |  |  |
| 8. Complacency (5C) | 1.39 | 0.91 | -.05** | .05** | -.05** | -.20** | .06** | -.24** | .03** |
|  |  |  | [-.07, -.02] | [.03, .07] | [-.07, -.03] | [-.22, -.18] | [.04, .08] | [-.26, -.22] | [.01, .05] |
|  |  |  |  |  |  |  |  |  |  |

*Note.* *M* and *SD* are used to represent mean and standard deviation, respectively. Values in square brackets indicate the 95% confidence interval for each correlation. The confidence interval is a plausible range of population correlations that could have caused the sample correlation (Cumming, 2014). * indicates *p* < .05. ** indicates *p* < .01.

**Table S10**: *Means, standard deviations, and correlations with confidence intervals for Annual influenza vaccination. “Do you get regular vaccinations against the seasonal influenza every year?”*

| Variable | *M* | *SD* | 1 | 2 | 3 | 4 | 5 | 6 | 7 |
| --- | --- | --- | --- | --- | --- | --- | --- | --- | --- |
|  |  |  |  |  |  |  |  |  |  |
| 1.Annual Influenza Vaccination | 0.37 | 0.48 |  |  |  |  |  |  |  |
|  |  |  |  |  |  |  |  |  |  |
| 2. Age | 48.32 | 17.76 | .25** |  |  |  |  |  |  |
|  |  |  | [.22, .27] |  |  |  |  |  |  |
|  |  |  |  |  |  |  |  |  |  |
| 3. Year | 1.50 | 0.50 | -.03 | -.13** |  |  |  |  |  |
|  |  |  | [-.05, .00] | [-.15, -.11] |  |  |  |  |  |
|  |  |  |  |  |  |  |  |  |  |
| 4. Confidence (5C) | 3.67 | 1.24 | .26** | -.00 | .04** |  |  |  |  |
|  |  |  | [.24, .29] | [-.02, .02] | [.02, .06] |  |  |  |  |
|  |  |  |  |  |  |  |  |  |  |
| 5. Constraints (5C) | 1.62 | 1.19 | -.11** | -.19** | .02* | -.00 |  |  |  |
|  |  |  | [-.14, -.09] | [-.21, -.17] | [.00, .04] | [-.02, .02] |  |  |  |
|  |  |  |  |  |  |  |  |  |  |
| 6. Coll. Responsibility (5C) | 4.68 | 0.84 | .07** | -.05** | .06** | .14** | -.03** |  |  |
|  |  |  | [.05, .10] | [-.07, -.03] | [.04, .08] | [.12, .16] | [-.05, -.01] |  |  |
|  |  |  |  |  |  |  |  |  |  |
| 7. Calculation (5C) | 3.91 | 1.37 | -.06** | .12** | -.03** | -.15** | -.03** | -.04** |  |
|  |  |  | [-.08, -.03] | [.10, .14] | [-.05, -.01] | [-.17, -.13] | [-.05, -.01] | [-.06, -.02] |  |
|  |  |  |  |  |  |  |  |  |  |
| 8. Complacency (5C) | 1.39 | 0.91 | -.10** | .05** | -.05** | -.20** | .06** | -.24** | .03** |
|  |  |  | [-.12, -.07] | [.03, .07] | [-.07, -.03] | [-.22, -.18] | [.04, .08] | [-.26, -.22] | [.01, .05] |
|  |  |  |  |  |  |  |  |  |  |

*Note.* *M* and *SD* are used to represent mean and standard deviation, respectively. Values in square brackets indicate the 95% confidence interval for each correlation. The confidence interval is a plausible range of population correlations that could have caused the sample correlation (Cumming, 2014). * indicates *p* < .05. ** indicates *p* < .01.

**Table S11**: *Means, standard deviations, and correlations with confidence intervals for the Intention to vaccinate against Influenza*

| Variable | *M* | *SD* | 1 | 2 | 3 | 4 | 5 | 6 | 7 |
| --- | --- | --- | --- | --- | --- | --- | --- | --- | --- |
|  |  |  |  |  |  |  |  |  |  |
| 1. Vaccination Intention Influenza | 0.46 | 0.50 |  |  |  |  |  |  |  |
|  |  |  |  |  |  |  |  |  |  |
| 2. Age | 48.32 | 17.76 | .20** |  |  |  |  |  |  |
|  |  |  | [.17, .23] |  |  |  |  |  |  |
|  |  |  |  |  |  |  |  |  |  |
| 3. Year | 1.50 | 0.50 | -.01 | -.13** |  |  |  |  |  |
|  |  |  | [-.04, .02] | [-.15, -.11] |  |  |  |  |  |
|  |  |  |  |  |  |  |  |  |  |
| 4. Confidence (5C) | 3.67 | 1.24 | .29** | -.00 | .04** |  |  |  |  |
|  |  |  | [.27, .32] | [-.02, .02] | [.02, .06] |  |  |  |  |
|  |  |  |  |  |  |  |  |  |  |
| 5. Constraints (5C) | 1.62 | 1.19 | -.00 | -.19** | .02* | -.00 |  |  |  |
|  |  |  | [-.03, .02] | [-.21, -.17] | [.00, .04] | [-.02, .02] |  |  |  |
|  |  |  |  |  |  |  |  |  |  |
| 6. Coll. Responsibility (5C) | 4.68 | 0.84 | .09** | -.05** | .06** | .14** | -.03** |  |  |
|  |  |  | [.06, .12] | [-.07, -.03] | [.04, .08] | [.12, .16] | [-.05, -.01] |  |  |
|  |  |  |  |  |  |  |  |  |  |
| 7. Calculation (5C) | 3.91 | 1.37 | -.07** | .12** | -.03** | -.15** | -.03** | -.04** |  |
|  |  |  | [-.09, -.04] | [.10, .14] | [-.05, -.01] | [-.17, -.13] | [-.05, -.01] | [-.06, -.02] |  |
|  |  |  |  |  |  |  |  |  |  |
| 8. Complacency (5C) | 1.39 | 0.91 | -.12** | .05** | -.05** | -.20** | .06** | -.24** | .03** |
|  |  |  | [-.15, -.10] | [.03, .07] | [-.07, -.03] | [-.22, -.18] | [.04, .08] | [-.26, -.22] | [.01, .05] |
|  |  |  |  |  |  |  |  |  |  |

*Note.* *M* and *SD* are used to represent mean and standard deviation, respectively. Values in square brackets indicate the 95% confidence interval for each correlation. The confidence interval is a plausible range of population correlations that could have caused the sample correlation (Cumming, 2014). * indicates *p* < .05. ** indicates *p* < .01.

**Figure S12**: 5C Scale Values for all items collected in 2016


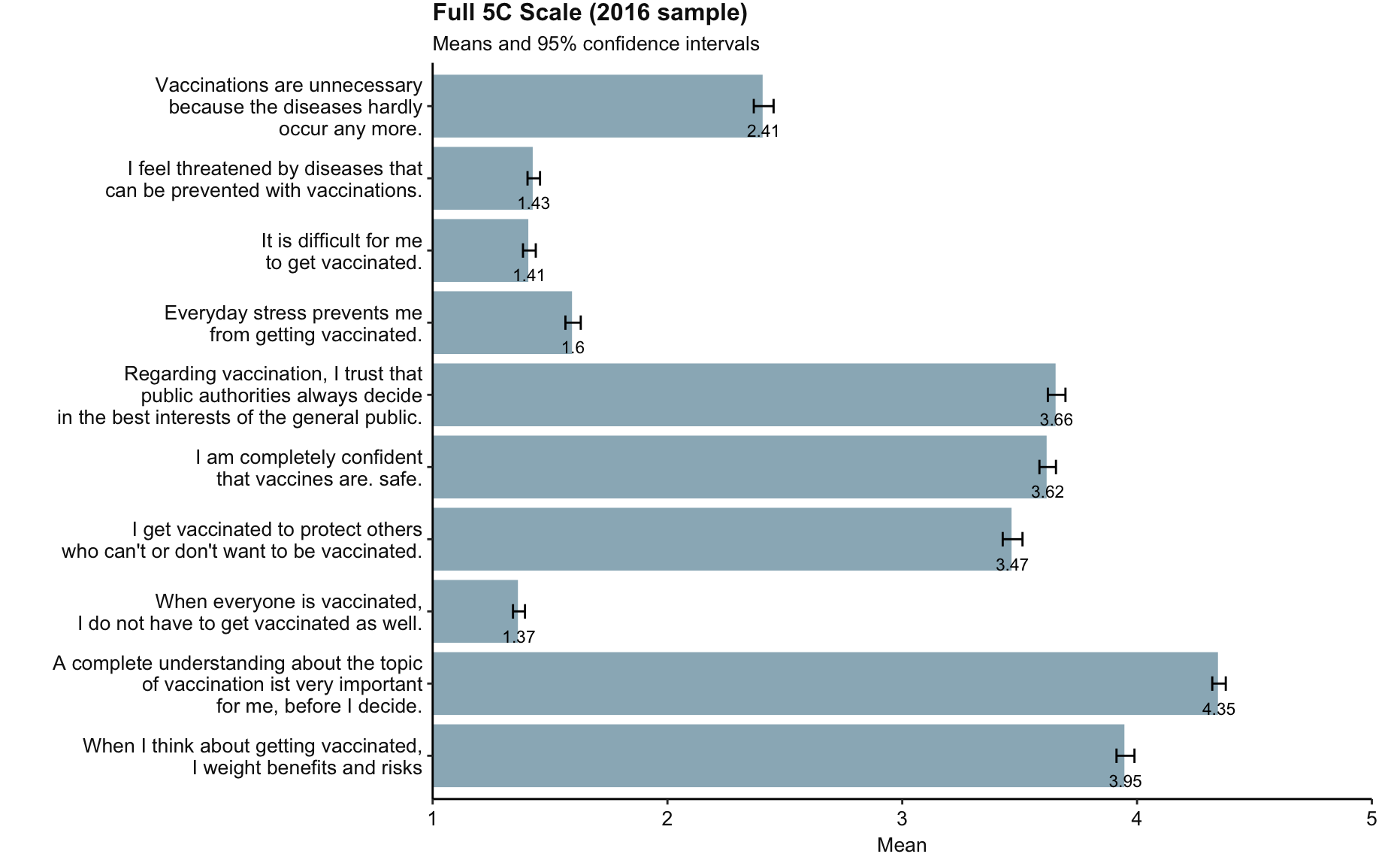

Supplement: Supplementary file 2 — Supplementary Material 2: Tables for Regression analyses. [file 12889_2024_18674_MOESM2_ESM.docx]
